# Sialendoscopy-Based Analysis of Submandibular Duct Papillae with a Proposal for Classification

**DOI:** 10.3390/jcm12031129

**Published:** 2023-01-31

**Authors:** Aleksandar Aničin, Anže Jerman, Jure Urbančič, Luka Pušnik

**Affiliations:** 1 Department of Otorhinolaryngology and Cervicofacial Surgery, University Medical Center Ljubljana, 1000 Ljubljana, Slovenia; 2 Department of Otorhinolaryngology, Faculty of Medicine, University of Ljubljana, 1000 Ljubljana, Slovenia; 3 Department of Maxillofacial and Oral Surgery, University Medical Center Ljubljana, 1000 Ljubljana, Slovenia; 4 Institute of Anatomy, Faculty of Medicine, University of Ljubljana, 1000 Ljubljana, Slovenia

**Keywords:** sialendoscopy, Wharton’s duct, classification, salivary gland papilla, salivary gland disorders, sialolithiasis, ductal stenosis, sialadenitis

## Abstract

Identifying a submandibular (Wharton’s) duct punctum often hinders sialendoscopy; however, there is a paucity of evidence on whether the appearance of Wharton’s duct papilla impacts the sialendoscopic procedure. A classification of Wharton’s duct papillae based on the macroscopic appearance, size of dilatation probes, and sialendoscopic approach was proposed herein. The classification describing four main types of papillae, A, B, C, and D, was prospectively evaluated on 351 Wharton’s duct papillae in 315 patients. For each papillae type, the demographic/clinical data, intraoperative complications, and time required for sialendoscope introduction were analyzed. Estuary-like papilla (type A) was commonly seen after spontaneous stone extrusion, had no intraoperative complications noted, and had the shortest time required for the sialendoscope introduction. Normal papilla (type B) was the most frequently observed papilla (48.1%), reflecting diverse underlying pathology, while difficult papilla (type C) was often associated with unfavorable anatomical variations of the mandible or floor of the mouth. Substantially closed papilla (type D) had the highest rate of intraoperative complications, namely, perforation with a false passage, and required the longest time for the sialendoscope introduction. In seven patients (2.0%), the entrance into the duct was feasible only through the fistula, while the sialendoscope introduction failed in eight patients (2.3%). In conclusion, the appearance of Wharton’s duct papillae may be influenced by the underlying pathology. Based on the proposed classification, papilla typology affects the duration of sialendoscope introduction and may influence the frequency of intraoperative complications.

## 1. Introduction

Sialendoscopy, a minimally invasive endoscopic procedure, has emerged as the preferred technique for diagnosing and treating inflammatory and obstructive salivary gland diseases. With visualization of the intraductal system, the technique enables precise recognition of stone formations, duct strictures, mucous plugs, mucosal membrane changes, and anatomical variations [1,2]. The procedure is not exclusively diagnostic, but is interventional; thus, it can be used for the extraction of salivary stones, salivary duct lavage, dilatation of stenotic segments, or instillation of various medications such as corticosteroids or antibiotics [3]. The sialendoscopic procedure begins with the localization of the salivary duct and orifice, followed by the sialendoscope introduction, ductal exploration, and therapeutic intervention [4]. Given the complexity of the procedure, mastery of the anatomy of the salivary glands, particularly the topography of ducts, is imperative [5].

The submandibular (Wharton’s) duct extends anteriorly and superiorly from both submandibular glands located in the submandibular space. The course of the duct against gravity, along with its curved and long anatomy, causes saliva stagnation and promotes salivary stone formation. The terminal part of the duct is located in the mouth floor and opens as an orifice of the Wharton’s duct papilla. The position of the duct and its 0.5–1.5 mm wide ostium is invariably symmetric, but quite unpredictable; consequently, Wharton’s duct papilla can occasionally be challenging to recognize [6,7,8]. Sánchez Barrueco et al. recently described a new entity called a lack of papilla distensibility, characterized by a fibrotic and nonelastic papilla where dilatation of the orifice is difficult to achieve, regardless of its localization [9]. Various pathologies, such as strictures or mouth oedema, can make the orifice of this fragile structure substantially smaller [10]. One report showed that in approximately 10% of the cases, even experienced operators have problems recognizing Wharton’s duct ostium, while inexperienced operators are incapable of correctly detecting the orifice in up to 30% of cases [5].

In some institutions, a substantial proportion of sialendoscopic procedures is performed under local anesthesia, which is unpleasant for the patient; thus, prompt localization of the papilla is imperative [5,11,12,13]. For easier recognition of the punctum, operators have conventionally employed different techniques. The visibility of the papilla can be enhanced with salivary gland massage or sialagogues (e.g., ascorbic acid or lemon juice) that promote saliva excretion [8,14], allowing saliva flow to be observed at the ostium. Nevertheless, the saliva reservoir is limited, especially in patients with hyposalivatory conditions such as Sjögren syndrome [15]. To overcome this problem, Luers et al. suggested the application of methylene blue near the caruncula [5]. The dye gives a good contrast compared to the reddish mouth floor. When the submandibular gland is massaged, the saliva washes out the dye near the caruncula and, thus, helps with the localization of the orifice. More frequently, the infiltration of sterile saline solution or local anesthetics, such as lidocaine, near the papilla is used for easier identification of the ostium and to achieve better stiffness of the papilla [1,16]. In addition to the previous techniques, the magnifying loupes or microscope can also be employed for easier recognition of the orifice [17,18]. After the initial papilla identification, the proceduralist generally dilatates the punctum using salivary probes of increasing sizes. Another technique that can ease the dilatation is the principle of Seldinger, whereas the dilatation is performed by introducing the smallest probe which is replaced by a titanium guide wire. The latter serves for the introduction of bougie which can help with the dilatation of stenotic segment [19].

The identification of Wharton’s duct orifice frequently impedes sialendoscopy [18]. Proceduralists mainly focus on techniques for easier recognition, and reports on papilla’s appearance are scarce [8,10]. It is known that papillae are highly variable in direction, shape, or localization. In addition, intraductal strictures can affect its appearance [10,20], thus affecting the cannulation of the orifice and the entire sialendoscopic procedure. Based on the long-term experience of the authors with sialendoscopy, different types of papillae that share a subset of commonalities have been observed; however, no precise description of Wharton’s duct papillae has yet been published. Accordingly, this study aimed to propose a classification of Wharton’s duct papillae based on macroscopic appearance, size of dilatation probes, and sialendoscopic approach. We further aimed to evaluate the types of papillae we have observed over the last six years according to the proposed classification and analyze demographic and clinical data, intraoperative complications, and time required for the sialendoscope introduction.

## 2. Materials and Methods

### 2.1. Ethical Approval

This study was approved by the National Medical Ethics Committee of the Republic of Slovenia (Permit no.: 0120-80/2017/4; approval date: 14 March 2017) and conducted per the Helsinki Declaration. The patients gave informed consent to be included in this study before the initial sialendoscopic procedure.

### 2.2. Proposed Classification of Submandibular Duct Papillae

**Figure 1:**
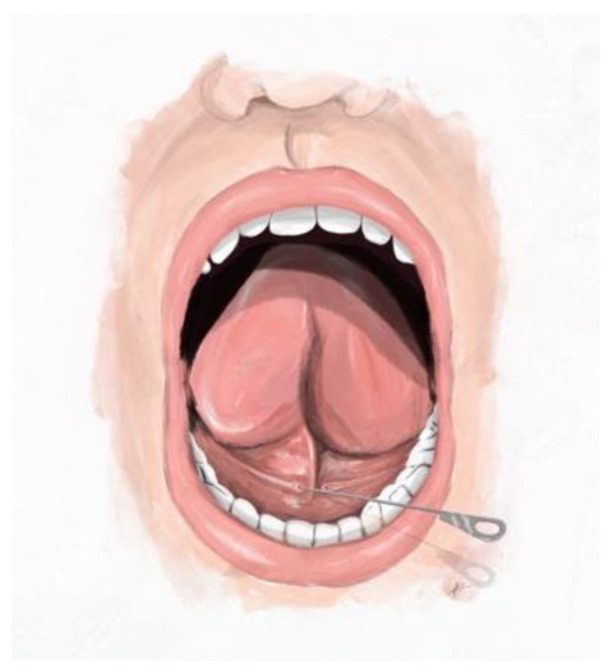
Insertion of the dilatation probe into the orifice of submandibular duct. The figure depicts the anatomy of the mouth floor, including two submandibular duct papillae. The dilatation probe is inserted into the right orifice of Wharton’s duct.

**Figure 2:**
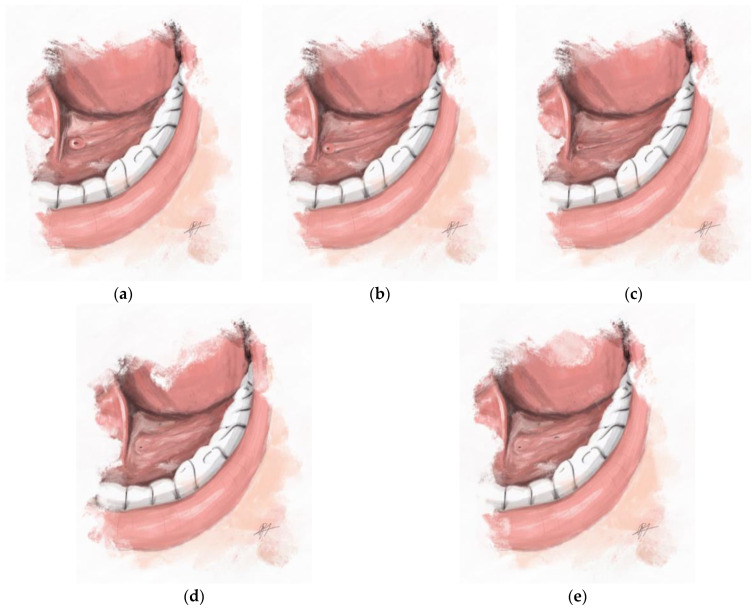
Graphical representation of different types of Wharton’s duct papillae. In (**a**), *easy* or *estuary-like papilla* (type A) can be seen. (**b**) depicts a *normal papilla* (type B) with evident orifice and papilla; and (**c**) demonstrates a *difficult papilla* (type C). (**d**,**e**) depict a *substantially closed papillae* (subtypes D_I/II_ and D_III_, respectively). In last two figures, the orifice is barely visible. An oval-shaped fistula can be noted near the orifice in the last figure.

The authors have observed different types of salivary gland papillae from experience. The herein proposed classification describing four main types of submandibular duct papillae was established on the basis of the macroscopic appearance of the papillae and the size of commercially available dilatation probes (Karl Storz, Tuttlingen, Germany). The fourth type of papilla was further divided into subtypes according to the sialendoscopic approach. The insertion of the dilatation probe is presented in Figure 1 and the proposed classification of papillae is depicted in Figure 2.

**Type A:***Easy* or *estuary-like papilla*. The papilla is markedly dilated, with an easy on-site probe insertion (diameter of initial probe: 00, 0, or thicker).

**Type B:***Normal papilla*. The papilla has an evident orifice, with saliva present when massaging the gland. The probe (diameter of initial probe: 000) can be inserted on-site without infiltration of anesthetic. The dilatation is straightforward, without returning to smaller diameters.

**Type C:***Difficult papilla*. The papilla does not have an evident orifice; the saliva is present; the infiltration of local anesthetics is often needed for recognition; and a rather difficult probe insertion (diameter of initial probe: 0000). 

**Type D:***Substantially closed papilla*. The papilla is barely visible or invisible. A small amount of saliva can be present at the orifice (or fistula). A probe of any size cannot be directly introduced into the main orifice, and the sialendoscope insertion through the natural orifice is not feasible. The type is further divided into the following subtypes according to the sialendoscopic approach:

**Subtype D_I_:** Papilla necessitating distal sialodochotomy; retrograde papilla dilatation is feasible.

**Subtype D_II_:** Papilla necessitating distal sialodochotomy; however, retrograde papilla dilatation is not feasible. 

**Subtype D_III_:** A sialendoscopy without papilla dilatation; an endoscope is inserted through the fistula (spontaneous or iatrogenic).

**Subtype D_F_:** Sialendoscopy failure. The entrance into the main duct is impossible; the distal sialodochotomy is not feasible.

### 2.3. Research Design

This study prospectively included patients who had sialendoscopy performed at the Department of Otorhinolaryngology and Cervicofacial Surgery of the University Medical Centre Ljubljana between March 2017 and December 2022. The inclusion criteria were the performance of at least one submandibular gland sialendoscopy for any of the following indications: obstructive sialadenitis (sialolithiasis or non-stone obstruction), recurrent *sine causa* sialadenitis, Sjögren syndrome, radioiodine-induced sialadenitis, or evaluation of intraductal mass. The exclusion criteria were the non-cooperation of the patient and the absence of the principal proceduralist (A.A.) during the sialendoscopy. The proceduralist had more than ten years of experience performing sialendoscopic procedures.

The demographic data, including sex, age, tobacco use, and alcohol consumption, were prospectively obtained during the examination. The patients had the type of papilla determined at the initial sialendoscopic procedure by a single operator (A.A.). The papilla type was determined according to the herein-presented classification. The patients who had the sialendoscopic procedure performed more than once served for the comparison within the same subjects. The lithiasis and duct strictures were recorded according to the LSD (lithiasis, stenosis, dilatation) classification [21]. In addition, intraoperative complications were noted.

The time required for the sialendoscope introduction was measured in five patients for each papilla type. The time of the sialendoscope introduction was defined as the time from the procedure commencement (i.e., the patient was under local anesthesia, prepared to open the mouth, and instant papilla identification) to the successful insertion of the sialendoscope into to orifice. The sialendoscopy was performed by the same operator (A.A.) in all patients.

### 2.4. Visualisation of Papilla, Papilla Dilatation, and Sialendoscope Introduction

**Figure 3:**
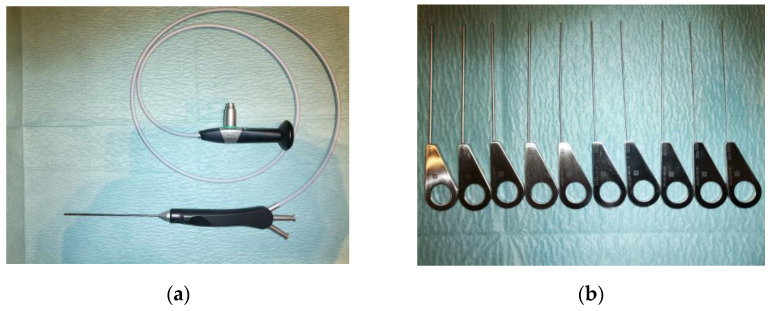
Sialendoscope and probes used for dilatation of papilla. (**a**) displays a sialendoscope (Karl Storz, Tuttlingen, Germany). In (**b**), dilatation probes (Marchal dilatator system, Karl Storz, Tuttlingen, Germany) with sizes in decreasing order are depicted (from size 6 to 0000).

Sialendoscopy was performed under local anesthesia in patients older than fifteen years. Younger patients and older patients with proximal salivary stones underwent general anesthesia. Patients were administrated 2% topical lidocaine on the mouth floor before commencing the procedure [22]. Initially, the proceduralist identified Wharton’s duct papilla using magnifying loupes. If the papilla was not evident, 1–2 mL of 2% lidocaine hydrochloride solution was injected submucosally near the expected orifice. The next step involved dilating the orifice using the commercially available salivary probes of increasing diameter (Marchal dilatator system, Karl Storz, Tuttlingen, Germany). The probes were available in sizes from 0000 to 6, as presented in Figure 3. The dilatated orifice served for the sialendoscope introduction. The sialendoscopes type Erlangen or type Marchal (Karl Storz, Tuttlingen, Germany) with an external diameter of 0.89 mm, 1.1 mm, 1.3 mm, or 1.6 mm were used. When the sialendoscope was inserted through the papilla, the salivary duct was irrigated with the 0.5% lidocaine hydrochloride solution to allow proper visualization on the screen. If the salivary probes with the diameter 0000 could not be inserted, the distal submandibular sialodochotomy was indicated, and after the sialendoscopic intervention, a retrograde approach through the papilla was evaluated [23]. If the sialodochotomy was not successful, the sialendoscope could not be introduced; thus, the sialendoscopy was defined as not feasible.

### 2.5. Statistical Analysis

Descriptive statistics were obtained to describe the characteristics of the papilla types. Numeric variables between the groups were evaluated by one-way analysis of variance (ANOVA) followed by Tukey’s post hoc tests. A chi-squared test was used to compare gender, tobacco use, and alcohol consumption between the groups. Statistical analysis was performed using GraphPad Prism 9 (GraphPad Software Inc., San Diego, CA, USA). *p* value < 0.05 was considered to indicate statistical significance. Data are presented as mean ± standard deviation (SD), ratio, or percentage when appropriate.

## 3. Results

The analysis of Wharton’s duct papillae during 351 sialendoscopic interventions is presented (left side, n = 151; right side, n = 200). Thirty-two (9.1%) of the procedures were repeat interventions, and four interventions were performed bilaterally (1.1%). There was a slight male preponderance in the analyzed population (53.6%), with a male-to-female ratio of 1.2:1. The mean age of the patients at the time of the sialendoscopic procedure was 46.3 ± 17.7 years.

**Figure 4:**
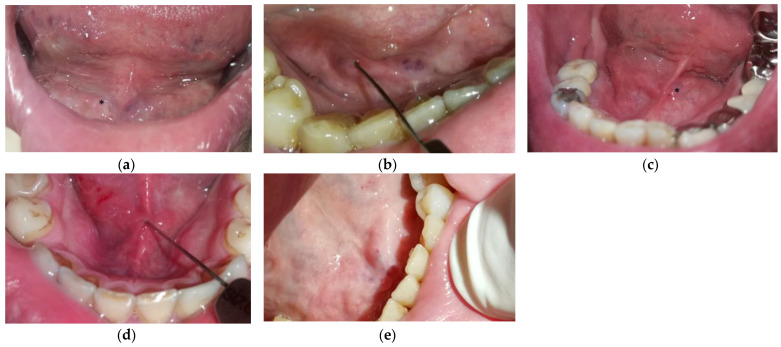
Submandibular duct papillae. (**a**) displays an *estuary-like papilla* (type A) on the patient’s right side before inserting the dilatation probes (asterisk *). The following two figures demonstrate *normal papilla* (type B) in two patients. In (**b**), the dilatator is inserted into the papilla, while the patient’s left papilla (asterisk *) is already dilatated in (**c**). (**d**) displays a *difficult papilla* (type C) with the dilatator inserted. In this patient, the narrow mandibula and mandibular torus hindered the insertion of dilatation probes. (**e**) shows one visible papilla on the patient’s left side, while *substantially closed papilla* (type D) on the patient’s right side cannot be visualized.

Based on the proposed classification, most papillae were classified as type B (48.1%). Approximately one-sixth of the papillae were classified as type A (17.7%), one-sixth as type C (17.4%), and one-sixth as type D (17.0%). The precise distribution of papilla types is presented in Table 1. Figure 4 depicts different papillae types observed during the interventions. There were no statistically significant differences between the groups in mean age, gender distribution, excessive alcohol consumption, or tobacco use (*p* > 0.05).

The most common indication for performing the sialendoscopic intervention was sialolithiasis (78.1%). Other indications for the procedure in descending order were: ductal stenosis (17.1%), recurrent *sine causa* sialadenitis (3.1%), Sjögren syndrome (0.9%), radioiodine-induced sialadenitis (0.6%), and intraductal mass evaluation (0.3%). Sialolithiasis was the most common indication for sialendoscopy in all groups except in patients with papilla type D_II_. The precise distribution of indications is presented in Table 2. All patients with sialolithiasis had an ultrasound performed before the sialendoscopy. In six patients with long and narrow stones, the sialoliths were missed; thus, the final diagnosis was set during the sialendoscopic procedure. According to the LSD classification, most patients with ductal stenosis were classified as S2 (66.7%). One-sixth of the patients with ductal stenosis were classified as S1 (16.7%) and one-sixth as S3 (16.7%). The patients with sialolithiasis were most frequently classified as L3a (34.6%), L3b (23.2%), or L1 (23.0%). Approximately one-fifth of the patients with sialolithiasis were classified as L2a or L2b (19.2%). In type A papillae, most patients with sialolithiasis (44.6%) were classified as L1.

When comparing the type of Wharton’s duct papillae of the same patients, in eighteen out of thirty-two interventions (56.3%), a different type of papillae was observed during the subsequent intervention. The mean time between the first and subsequent procedure was 8.1 ± 7.3 months. All the patients had the same indication for repeated intervention. When considering solely the patients with sialolithiasis, the papilla during the subsequent intervention was classified as the same in 46.2% of interventions. In another 46.2% of interventions, the papilla was more evident and/or the dilatation could be initiated with a wider initial probe. In all four patients with stenosis, the papilla during the subsequent procedure required thinner dilatation probes.

Intraoperative complications were noted in 14 patients (4.0%). The complication observed during sialendoscopic intervention were duct perforation with false passage (n = 11; 3.1%), minor bleeding (n = 1; 0.3%), and damage to the lingual nerve (n = 2; 0.6%). Types D_II_ had the most complications noted (20.0%), while no intraoperative complications were observed in types A and D_F_. Types B, C, and D_I_ had 3.0%, 4.9%, and 3.3% rates of intraoperative complications, respectively. In type D_III_, the introduction of the sialendoscope was possible solely through the fistula, which was located on the mouth floor. In one patient with this papilla type (16.7%), the lingual nerve was injured. 

The time required for sialendoscope introduction was measured in a group of thirty patients. The time for the sialendoscope introduction of each group is presented in Table 3. The longest time was required for the introduction of the sialendoscope into the orifice of papillae D_I_ and D_II_, while type A had the shortest time. In type C, unfavorable anatomical variants often hindered the sialendoscope insertion. There was a statistically significant difference between the groups (*p* = 0.0001).

## 4. Discussion

In this study, a classification of Wharton’s duct papillae was proposed based on the appearance of the papillae, dilatation probes, and sialendoscopic procedure. The proposed classification describes four types of papillae. With prospective analysis of the patients, we found that the type of papilla was not dependent on demographic data; nonetheless, individual types of papillae might be related to specific underlying pathologies. Additionally, statistically significant differences were noted between different types of papillae and the time required for the sialendoscope introduction.

The histological studies on Wharton’s duct and papilla are scarce, and most existing studies were performed on cadavers [24,25]. The main duct consists of epithelium and connective tissue, predominantly the elastic fibers that contribute to the elasticity of the duct. The parallel distributed smooth muscle cells, dispersed throughout the connective tissue, do not enable proper constriction; hence, the duct/papilla does not have a sphincter function or role in salivary flow regulation [24]. The specific structure of the ductal wall ensures continuous salivary flow up to the orifice. At the opening of Wharton’s duct, there is a thin epithelial layer with an abundance of blood vessels [25]. Different pathological conditions that result in duct strictures may alter the orifice. The strictures mainly consist of collagen and abundant inflammatory infiltrate [8,26], providing a histological substrate for certain types of the papilla. 

The herein-proposed classification describes four distinct types of papillae showing key differences in the macroscopic appearance of papillae, the size of dilatation probes, and the outcome of sialendoscopic procedures, including the ability to retain intact salivary outflow tract. There were no demographic associations recognized. Therefore, we could not show any differences in age or gender distribution between the groups. Moreover, the groups did not differ in alcohol consumption or tobacco use, indicating that toxins that irritate the mucous membrane or cause oral epithelial dysplasia do not directly impact the papilla type [27]. Intuitively, the type of papilla should depend at least on the underlying pathology to some extent. Supporting the influence of recognized pathologic findings, in type A of the papilla, sialolithiasis was present in more than 90% of the cases, while in type D_II_, stenosis was the main indication for sialendoscopy in two-thirds of the patients. Also supporting our findings, Sánchez Barrueco et al., who analyzed patients with the lack of papilla distensibility, showed that tobacco use or alcohol consumption were not predisposing factors for this condition. Conversely, they have noted that older female patients are significantly more likely to be diagnosed with this condition; however, their study additionally included patients with parotid gland disorders [9]. 

The integrity of the submandibular duct and papilla has an important role in the pathophysiology of obstructive disease; hence, a thorough dilatation is a necessary part of a sialendoscopic procedure [10,25]. In type A papillae, the dilatation of the orifice was not always necessary. According to the LSD classification, floating stones were often observed [21]. As the stones accumulating around the opening area can be extruded naturally [18], we hypothesize that this could cause the dilatation of the orifice within this type of papilla. Accordingly, the patients within this group frequently reported spontaneous stone extrusion. Type A papilla was often seen at the subsequent sialendoscopic procedure of the patients who had the salivary stones removed in previous interventions. Therefore, the type of papilla supposedly also depends on the previously performed sialendoscopic procedure. Almost half of the patients with sialolithiasis who had sialendoscopy performed more than once had the papilla type that required larger dilatation probes at the subsequent procedure. Less than half of the patients had the same type of papilla noted during the second intervention, indicating possible papilla change over time.

Papilla type B was the most commonly observed papilla, and the indications for sialendoscopy within this group were diverse. In type C of the papilla, the insertion of the sialendoscope was rather difficult, mainly attributable to the unfavorable anatomical variants not exclusively related to the papilla itself, e.g., papilla near the alveolar ridge, narrow jaw, overhanging alveolar ridge or presence of mandibular torus. Consequently, this prolonged the time required for the sialendoscope introduction. 

Papilla type D showed a higher rate of intraoperative complications, namely, the duct perforation with a false passage. This was expected as this papilla type required the most invasive procedures to be dilatated. We hypothesize that the inflammatory process in the ducts contributed to the wall change and consequently led to a higher risk of perforation [8,20]. Accordingly, we would recommend the use of stents, especially in papilla type D_I_ (and type C), or in any other type in the simultaneity of other events, e.g., when the stenosis is present proximal to the papilla, when the salivary stone is removed via retropapillary approach, or in the case of false passage formation near the papilla. Subtypes D_I_ and D_II_ also required the longest time for the sialendoscope introduction. In both subtypes, the direct insertion of the sialendoscope into the orifice was not feasible; therefore, distal sialodochotomy with a retrograde approach was required. Subtype D_II_ had an impossible retrograde papillary dilatation, indicating more extensive or long duct narrowing present near the papilla. This is in accordance with Koch et al., who reported that stenoses are located near the papilla in almost two-thirds of the cases [20]. It is noteworthy that non-evident papilla does not inevitably indicate salivary duct stenosis. In an inflamed duct of large sialolith, the mouth floor can become edematous, making the orifice hardly visible [5]. However, the recognition of one stenosis near the papilla is essential, as the location has a significant impact on the treatment choice. The centrally located stenoses are more likely to be treated with the sialendoscopy, while in the stenoses near the papilla, other surgical techniques (e.g., marsupialization or ductostomy) are required [28]. Alternative to the herein-presented dilatation of papillae is the Seldinger technique [4], which can be considered in some cases as it enables the entrance and dilatation of the main duct; nonetheless, this technique is rarely used in our institution and, thus, not taken into account in the current papillae classification. Again, considering the aforementioned study by Sánchez Barrueco [9], where authors disclose that lack of papillae distensibility of the submandibular gland was most commonly noted in the patients with sialolithiasis, this could somehow overlap with the papilla types D_I_ and C.

We acknowledge that this study had some limitations. First, the papillae were assessed in a group of patients with salivary outflow pathology. Therefore, the distribution of papillae cannot be easily generalized. As the classification requires dilatation probes, assessing the papilla in healthy individuals would be unethical. A second limitation is a relatively small sample subdividing into individual smaller groups, which were more difficult or impossible to compare with statistical tests. It would be imperative in the future to expand the sample size with comparative studies. A third limitation is that no inter-rater comparison was conducted. Due to ethical reasons, the same patients could not be evaluated by two different operators simultaneously by inserting the salivary probes. In addition, inserting dilatators into already dilatated papilla could also alter the result. The last limitation is the absence of comparison between intervened and contralateral papillae; thus, future studies should inquire whether papilla typology is predominantly acquired or rather constitutionally determined.

## 5. Conclusions

In conclusion, different types of Wharton’s duct papillae were not dependent on demographic data such as age, gender distribution, tobacco use, or alcohol consumption; however, individual types of papillae might be related to specific underlying pathologies. The results illustrate the clinical validity of the proposed papilla classification and its potential as a research and clinical tool for diagnosing and treating salivary duct pathologies.

## Figures and Tables

**Table 1:** Analysis of demographic data.

Type of Papillae	Procedures ^1^ N (%)	Male-to-Female Ratio	Mean Age ± SD [Years] ^2^	Heavy Alcohol Consumption ^a,1^ N (%)	Tobacco Use ^b,1^ N (%)
A	62 (17.7)	1.4:1	50.3 ± 15.8	2 (3.2)	15 (24.1)
B	169 (48.1)	1.1:1	46.9 ± 14.7	9 (5.3)	38 (22.5)
C	61 (17.4)	1.7:1	42.0 ± 17.2	2 (3.2)	21(34.4)
D_I_	30 (8.4)	1.5:1	52.8 ± 24.6	2 (6.7)	4 (13.3)
D_II_	15 (4.2)	0.3:1	48.4 ± 17.8	0 (0.0)	4 (26.7)
D_III_	7 (2.0)	0.5:1	41.7 ± 3.3	0 (0.0)	1 (14.3)
D_F_	8 (2.3)	1:1	47.1 ± 20.9	1 (12.5)	3 (37.5)

SD—standard deviation; N (%)—number of interventions with the percentage according to the type of papilla. ^a^ within the group, based on the anamnesis, whereas heavy alcohol consumption was defined as ≥7 drinks per week for women or ≥ 14 drinks for men. ^b^ within the group, based on the current anamnesis—yes or no. All data are non-significant at *p* > 0.05 (^1^ chi-squared test or ^2^ one-way ANOVA).

**Table 2:** Analysis of indications for the sialendoscopy.

Type of Papillae	Sialolithiasis N (%)	Stenosis N (%)	Recurrent *Sine Causa* Sialadenitis N (%)	Sjögren Syndrome N (%)	Radioiodine-Induced Sialadenitis N (%)	Intraductal Mass N (%)
A	56 (90.3)	6 (9.7)	0 (0.0)	0 (0.0)	0 (0.0)	0 (0.0)
B	136 (80.5)	20 (11.8)	7 (4.1)	3 (1.8)	2 (1.2)	1 (0.6)
C	44 (72.1)	16 (26.2)	1 (1.7)	0 (0.0)	0 (0.0)	0 (0.0)
D_I_	23 (76.7)	7 (23.3)	0 (0.0)	0 (0.0)	0 (0.0)	0 (0.0)
D_II_	5 (33.3)	10 (66.7)	0 (0.0)	0 (0.0)	0 (0.0)	0 (0.0)
D_III_	6 (100.0)	0 (0.0)	0 (0.0)	0 (0.0)	0 (0.0)	0 (0.0)
D_F_	4 (50.0)	1 (12.5)	3 (37.5)	0 (0.0)	0 (0.0)	0 (0.0)

N (%)—absolute number of affected patients with the percentage based on the type of papilla group.

**Table 3:** Time required for the introduction of the sialendoscope.

Type of Papillae	Sialendoscope Introduction Time [min]
A	1.9 ± 1.0
B	3.8 ± 1.8
C	7.1 ± 3.8
D_I_	24.1 ± 6.3
D_II_	23.3 ± 4.1
D_III_	2.9 ± 0.9
D_F_	NP

Data are presented as mean ± standard deviation. NP—the introduction of the sialendoscope was not feasible. The differences between the groups are statistically significant at *p* < 0.0001 (one-way ANOVA).

## Data Availability

The data presented in this study are available on request from the corresponding author.
